# Modulation of peripheral T-cell function by interleukin-7 in rheumatoid arthritis

**DOI:** 10.1186/s13075-014-0511-3

**Published:** 2014-12-23

**Authors:** Sarah M Churchman, Jehan J El-Jawhari, Agata N Burska, Rekha Parmar, Vincent Goëb, Philip G Conaghan, Paul Emery, Frederique Ponchel

**Affiliations:** Leeds Institute of Rheumatic and Musculoskeletal Medicine, St James’s University Hospital, Beckett Street, Leeds, LS9 7TF UK; Department of Rheumatology, University Hospital of Amiens, University of Picardie Jules Verne, Chemin du Thil, Amiens, 80000 France; Leeds Institute of Rheumatic and Musculoskeletal Medicine, Leeds Musculoskeletal Biomedical Research Unit, Chapel Allerton Hospital, Chapeltown Road, Leeds, LS7 4SA UK

## Abstract

**Introduction:**

Interleukin-7 (IL-7) is a cytokine essential for T-cell lymphopoiesis, survival and polarization with an emerging role in autoimmunity. We previously demonstrated reduced levels of circulating IL-7 in rheumatoid arthritis (RA), although high amounts are expressed in joints, suggesting differences between systemic and synovial effects. We observed healthy levels of IL-7 in 48% of RA patients in clinical remission (CR) and aimed to investigate the consequences of IL-7 deficiency on T-cell responses.

**Methods:**

We used RA patients with active disease and in CR presenting various levels of IL-7, to investigate its modulatory effects on T cells by analysing responses to phyto-haemagglutinin (PHA), expression of polarization or survival factors, or suppression by regulatory T cells (Tregs).

**Results:**

IL-7 levels were normal (>10 pg/ml) in 48% of RA patients in CR. Amongst 63 CR patients followed up for 18 months, lack of IL-7 recovery was observed in 13 out of 15 (86%) patients experiencing relapse but only 11 out of 48 (23%) of those who did not (*P* = 0.0002). Binary regressions showed high significance for below normal IL-7 levels for self-reported maternal family history of arthritis (odds ratio (OR): 7.66, *P* = 0.006) and a trend for smoking (OR: 3.33, *P* = 0.068) with no further demographic or clinical associations. Serum IL-7 correlated with restored CD4^+^T-cell response to PHA (rho = 0.879); this was not related to an increase in T-cell proliferation capacity or expression of survival factors B-cell lymphoma 2 (BCL2) and BCL2-associated protein X (BAX). Expression of Th1 polarization factor (*TBET*) was also dependent on exposure to IL-7 *in vivo* (rho = 0.600). In contrast CD25^high^Tregs’ response to PHA was not affected by *in vivo* IL-7, but their suppression capabilities were related to circulating IL-7 (rho = 0.589). Co-stimulation with IL-7 (mimicking the joint environment) increased responsiveness of CD4^+^T-cells to PHA, lowering the ability of CD25^high^Tregs to suppress them.

**Conclusions:**

Our data demonstrate that IL-7 has a critical role in modulating T-cell function *in vivo,* possibly explaining opposing effects observed systemically and in the joint. Lack of IL-7 recovery in CR by maintaining a suppressed immune system may be a determinant factor in the occurrence of relapse.

**Electronic supplementary material:**

The online version of this article (doi:10.1186/s13075-014-0511-3) contains supplementary material, which is available to authorized users.

## Introduction

The exact pathogenesis of rheumatoid arthritis (RA) remains uncertain, but autoimmune processes are clearly relevant as evidenced by major histocompatibility complex (MHC) linkage [1,2], auto-antibodies (rheumatoid factor (RF)) and other antigenic specificities [3]. Lymphocyte infiltration into the synovium is an important feature of the disease. A ‘T-centric’ hypothesis however, presents with a number of paradigms, notably activated T cells should be central to this model, but appear predominantly anergic in the blood of RA patients [4]. Recently, the genetic risk [5–8] associated with RA has largely implicated T-cell biological processes, reactivating the interest in this cell type.

Our work on T cells in RA over several years has suggested that particular subsets are lost in RA notably recent thymic emigrants [9,10] naïve and memory CD4^+^T cells, [9] regulatory T cells (Tregs) [11], and compensated by the presence of abnormal subsets (inflammation related cells, IRCs) [9]. Some of these abnormalities have been shown to be associated with relapse following disease-modifying anti-rheumatic drug (DMARD)-induced remission [12], predict safe discontinuation of a therapeutic anti-tumour necrosis factor (TNF) agent [13] and more recently predict methotrexate (MTX)-induced remission in early RA [14] as well as progression towards RA in anti-citrullinated protein antibody-positive (ACPA+) at risk individuals (unpublished observation presented at the European League Against Rheumatism (EULAR) Congress 2013 and the European Workshop for Rheumatology Research (EWRR) 2014). We also associated reduced levels of interleukin-7 (IL-7) in the bone marrow, thymus and blood with profound and persistent lymphopenia in RA post chemotherapy [10,15–17] and showed normalization of circulating IL-7 levels in approximately 50% of RA patients in clinical remission (CR) defined by disease activity score (DAS) <2.6 [12].

Recent studies demonstrated that IL-7 is overexpressed in several autoimmune diseases [18,19]. It primarily acts on T cells inducing T helper cell (Th)1- and Th17-associated cytokine secretion [20,21], dendritic cell (DC) activation with the production of T-cell differentiating factors, chemokines, adhesion/co-stimulatory molecules and T-cell-dependent activation of macrophages (recently reviewed by Bikker and colleagues [22]). IL-7 also coordinates ectopic lymphoid formation [23–25] as well as T-cell-driven osteoclastogenesis [26,27].

IL-7 is a cytokine of the IL-2 family. It is either secreted into the circulation or presented in solid tissue by heparan sulfate and fibronectin on cell surfaces [28]. The IL-7 receptor (IL-7R) is expressed on all circulating CD4^+^ and CD8^+^ T cells and natural killer (NK) cells, but not on mature human B cells. IL-7 has now been associated with the pathogenesis of RA. Early data showed that IL-7 is expressed at higher levels in RA synovial tissues than in osteoarthritis (OA) [15,29] and its expression is related to local inflammation, measured by either anti-CD68 immunohistochemistry [29] or by arthroscopic inspection [15,30]. Fibroblasts isolated from RA synovium spontaneously produce IL-7 [31] in direct relation to their level of exposure to inflammation *in vivo* [30] and a significant increase was detected upon their stimulation with IL-1β or TNF-α. Synovial, but not blood T cells, respond to IL-7 by spontaneous proliferation [31,32]. IL-7 stimulation increases the production of TNF-α by synovial fluid mononuclear cells (SFMCs) and more specifically of TNF-α and interferon (IFN)-γ by CD4^+^T cells [33]. SFMCs pre-incubated (primed) with IL-7 for five days produced the same effects on CD4^+^T cells in the absence of further stimulation with IL-7 [33]. In contrast, we and others showed that both peripheral blood T cells from RA patients and healthy controls were unresponsive to IL-7 stimulation alone at physiological concentrations [10,32]. These results suggest possible differences in T-cell function between the periphery and the joint, where *in vivo* levels are respectively low and high [10,17].

This observation prompted us to hypothesize that IL-7 may be responsible for some of the differences in T-cell features between active and remitting disease. We had already demonstrated a direct correlation between *in vivo* levels of circulating IL-7 and the functional capacity of the thymus [10] and showed that peripheral blood mononuclear cells (PBMCs) from RA patients in CR exhibited increased responsiveness to phyto-haemagglutinin (PHA) *in vitro,* in direct relation to their *in vivo* exposure to IL-7 [15]. Since approximately half of RA patients in CR showed restored normal levels of circulating IL-7 [10], this presented an opportunity to evaluate the effect of normal and reduced *in vivo* exposure to IL-7 in active and remitting RA.

Using groups of patients either with active RA or in CR, we observed that the lack of recovery of IL-7 in CR was associated with relapse in the short term. To explain this observation, we showed that *in vivo* exposure to different levels of IL-7 has a profound effect on T-cell response to PHA stimulation tested *in vitro.* This was not directly related to a change in the proliferative capability of effector CD4^+^T cells in reaction to IL-7 or to the expression of survival factors. Increased expression of the Th1 (but not Th2) polarization transcription factor was also observed in relation to IL-7. In contrast to effector cells, regulatory T-cell proliferation was not affected but their suppression capabilities were directly related to exposure to IL-7 *in vivo*.

## Methods

### Participants

Ethical approval was obtained from the Leeds Teaching Hospitals NHS Trust Ethics Committee, and informed consent was obtained from each participant. RA was diagnosed using the American College of Rheumatology (ACR) 1987 criteria. Patients in the early stages of RA (<2 symptomatic years) were DMARD-naïve (DN) and divided into those with less than 6 months’ symptom duration (<6 m DN) (n = 127) and those with 6 to 24 months’ symptom duration (6 to 24 m DN) (n = 37). Long-lasting RA included DMARD-resistant patients (n = 55). RA patients in CR (n = 90) were defined using a clinical DAS28 < 2.6, median 1.6, range 0.64 to 2.58) sustained for at least 6 months; low-grade inflammation defined by C-reactive protein (CRP <10 mg/L, upper limit of the normal CRP range in the local population). Patients were on MTX (46%), sulphasalazine (20%), combination of both (23%) or no DMARDs (11%). The term ‘active’ RA refers to all RA patients except those in CR. Controls included healthy subjects (HC, n = 80) and other inflammatory disease (active colitis, psoriatic arthritis, ankylosing spondylitis, gout, reactive arthritis, n = 96) as well as OA, n = 19. Demographic data can be found in Additional file 1. There was no significant difference in age range and gender representation between groups.

### Measurement of cytokines

All cytokine levels were measured in sera and synovial fluid (SF) by enzyme-linked immunosorbent assay (ELISA) (R&D Systems, Abingdon, UK), according to the manufacturer’s instructions. The sensitivity of the IL-7 assay was 0.1 pg/ml and the range was 0.1 to 24 pg/ml. 1:3 dilution was used for serum and 1:5 for SF. For other ELISAs (R&D) the ranges for IL-2, IL-6, IFN-γ, TNF-α and transforming growth factor (TGF)-β1 were 31.2 to 2,000, 3 to 500, 7 to 1,000, 7 to 1,000 and 15 to 3,000 pg/ml, respectively.

### Immunohistochemistry and digital imaging scoring

Full details of this method can be found in Additional file 1. Briefly, slides from paraffin-embedded sections were dewaxed, washed and stained with mouse monoclonal IL-7 antibody (R&D Systems, MAB207). Universal probe and X-Cell Polymer HRP reagents (Menarini Diagnostics, Wokingham, UK) were applied, followed by 3′-diaminobenzidine. Slides were counterstained in haematoxylin, dehydrated by ethanol and mounted in di-n-butyle phthalate xylene. Quantification of IL-7 was performed electronically using 256 shades of DAB with anything above shade 50 considered positive for IL-7.

### Phenotypic analysis of T cells and Tregs

T cells and Tregs were phenotyped using naïve and memory cell surface markers as well as Foxp3 intracellular staining as previously described [14]. Naïve cells were defined as CD3^+^CD4^+^CD45RB^high^CD45RA^+^CD62L^+^ and IRCs as CD3^+^CD4^+^CD45RB^high^ CD45RA^+^CD62L^−^ and Tregs based on CD3^+^CD4^+^ and high expression of CD25 (CD25^high^) [34], with the addition of Foxp3^+^ and CD127^low^ [35,36]. All subsets were quantified as a percentage of all CD4^+^ T cells. Expression of CD127 was recorded using mean fluorescence intensity (MFI).

### Cell sorting

PBMCs were separated by centrifugation using Lymphoprep (Axis Shield Diagnostics Ltd, Huntingdon, UK) from 30 ml of heparin blood. Magnetic negative selection of CD4^+^T cells was performed as previously described [11]. CD4^+^T cells were labelled using anti-CD3-FITC (UCHT1, AbD Serotec, Kidlington, UK), and anti-CD4-PE-Cy5 (MT310, Dako, Ely, UK), anti-CD25-PE (CD25-3G10, Caltag Laboratories, Burlingame, CA, USA) [11]. CD4^+^CD25^−^ and CD4^+^CD25^high^ fractions were sorted. Both were assessed for purity using the antibody panel described above (CD3, CD4, CD25, CD127 and intracellular FoxP3) and results showed over 95% pure effector T cells or Tregs.

### Proliferation and suppression assays

Total CD4^+^T cells were negatively selected [11] and resuspended (10^4^/well) in RPMI 1640 medium supplemented with penicillin, streptomycin, glutamine (Invitrogen, Paisley, UK) and 10% human AB+ serum (Sigma-Aldrich, Poole, UK). Subsequent assays were performed in 96-well round-bottomed plates (200 μl/well) in the presence of an equal number of autologous mitomycin C-treated CD4-depleted PBMCs as antigen-presenting cells, in response to PHA (1 μg/ml, Sigma-Aldrich).

Proliferation of sorted CD4^+^CD25^−^ and CD4^+^CD25^high^T cells was compared following PHA stimulation (2 μg/ml), with or without the addition of IL-7 (10 ng/ml, R&D Systems) [11]. The ability of CD4^+^CD25^high^Tregs to inhibit CD4^+^CD25^−^T-cell proliferation was assessed in four-day co-culture experiments (1:1 ratio), in the presence of PHA (2 μg/ml), as previously described [11] with or without the addition of IL-7 (10 ng/ml).

### Real-time PCR quantification of TRECs

T-cell receptor excision circles (TRECs) were quantified using a real-time PCR-based assay as defined previously [37] using absolute quantification. DNA was extracted from each subset using a standard proteinase K digestion and phenol/chloroform extraction. The previous relative method was used to quantify TREC in total and naïve CD4 T cells [9,38,39]. TREC molecules were normalized against DNA concentration for cells sorted as Treg, effector and naïve as the cell number after sorting was very low.

- TREC-F-5′-CACCTCTGGGCTACGTGCTAG-3′,
- TREC-R-5′-GAACACATGCTGAGGTTTAAAGAGAAT -3′.

### Real-time PCR analysis of gene expression

RNA was extracted from PBMC and cDNA synthesized, as described previously [40]. Real-time PCR was performed using an ABI7700 sequence detection system (Applied Biosystems, Warrington, UK) in the presence of SYBR Green. Optimization of the real-time PCR reaction was performed according to the manufacturer’s instructions. Each gene transcript was normalized to the reference gene *GAPDH*. Primer sequences as follows:

- GAPDH-F-5′-AACAGGGACACCCACTCCTC-3′,
- GAPDH-R-5′-CATACCAGGAAATGAGCTTGACAA-3′,
- BCL-2-F-5′-GTGGAGAGCGTCAACCGG-3′
- BCL-2-R-5′-GGTTCAGGTACTCAGTCATCCACA-3′,
- BAX-F-5′-GCCACTCCTCTGGGACCC-3′
- BAX-R-5′-ACGCATTATAGACCACATCTGATG-3′,
- T-BET/TBX21-F-5′- TCATTGCCGTGACTGCCTAC-3′,
- T-BET/TBX21-R-5′- TGTACATGGACTCAAAGTTCTCCC-3′,
- GATA3-F-5′- ACTGGAGGACTTCCCCAAGAAC-3′,
- GATA3-R-5′-GGCGAGATGTGGCTCAGG-3′

### Statistical analysis

Linear variables were not normally distributed amongst groups of controls or patients; therefore non-parametric tests were used throughout. Spearman’s rank correlation coefficient was used to correlate two variables. Mann-Whitney *U* test for two independent samples was used to compare groups and Kruskal-Wallis for comparisons between more than two independent samples where appropriate. *P* values were adjusted for significance using Dunn’s multiple comparison test. IBM SPSS Statistics 21 software was used.

## Results

### IL-7 levels in disease and controls

**Figure 1:**
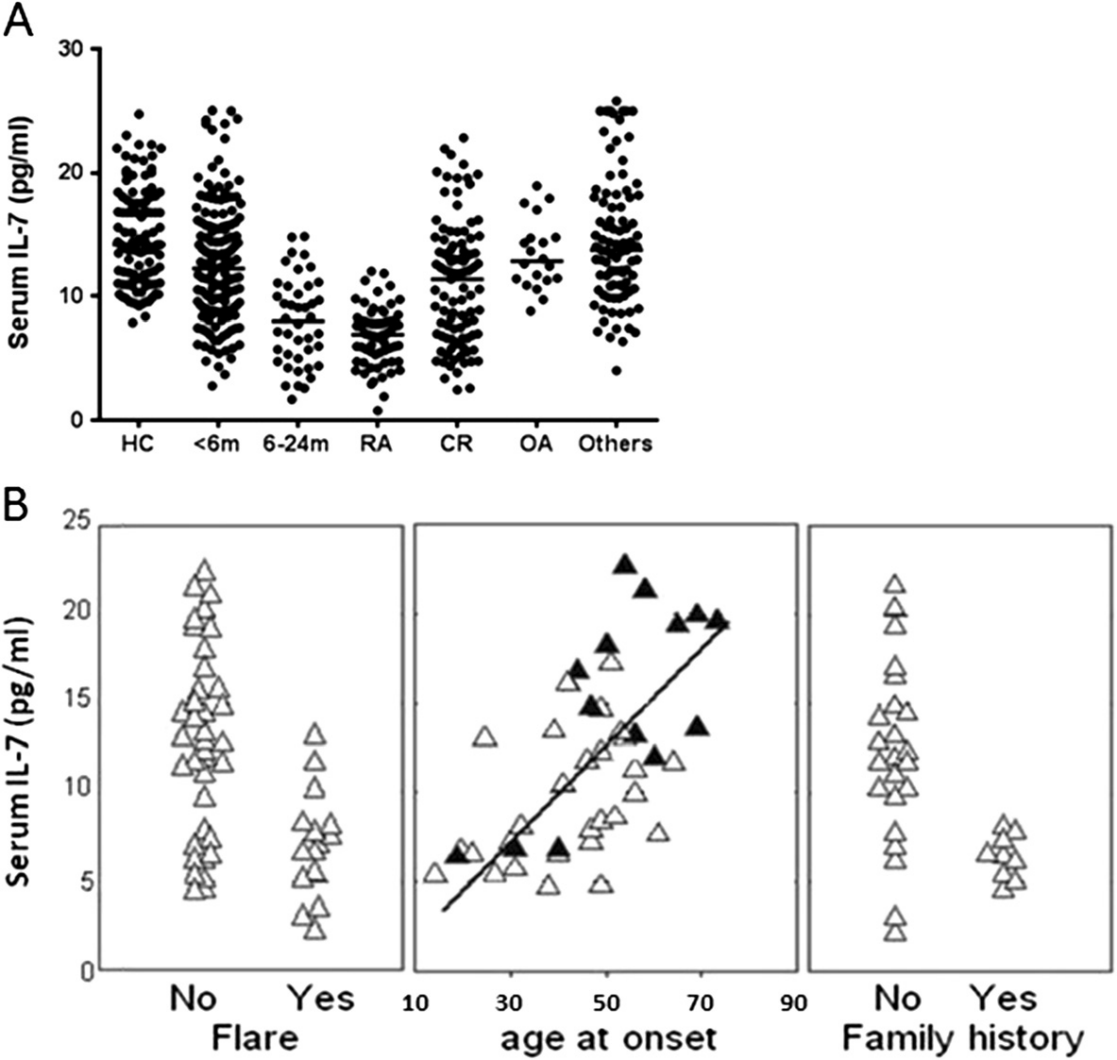
**IL-7 measures in controls and RA patients. (A)** Circulating levels of interleukin (IL)-7 in healthy controls (HC, n = 80), disease-modifying anti-rheumatic drug (DMARD)-naïve (DN) rheumatoid arthritis (RA) with less than 6 months’ symptom duration (<6 m, n = 127), 6 to 24 months’ symptom duration RA (also DN, 6 to 24 m, n = 37), established long-lasting RA (RA, n = 55), clinical remission (CR, n = 90) osteoarthritis (OA, n = 19) and other disease controls (n = 96). All RA groups (<6 m DN, 6 to 24 m DN and RA) showed significantly reduced IL-7 levels (all *P* <0.005) compared to HC. **(B)** The ability to recover normal levels of IL-7 in CR was examined with respect to different clinical and demographic parameters. Lack of IL-7 recovery was significantly associated with patients in CR destined to flare within an 18-month follow-up period (*P* = 0.072) as well as with patient self-reported maternal family history of arthritis (*P* = 0.003). Age at disease onset was directly related to recovered IL-7 levels (rho = 0.539, *P* = 0.001). Black triangles are males and open triangles are females.

Circulating levels of IL-7 have been extensively studied in HC and active RA [10,15,17] Median IL-7 levels were 14.2 pg/ml in HC (Figure 1A, n = 80, range 7.9 to 30.8 pg/ml with a 95% of values above 10 pg/ml, in agreement with Goëb *et al*. [17] and a review of 17 manuscripts incorporating approximately 400 healthy donors [15]) and 7.6 pg/ml in active RA (with >2 years duration, range 1.9 to 10.9 pg/ml) on several DMARD treatment regimes. Early, DN RA with <6 months symptom duration (<6 m DN) showed reduced levels of IL-7 (median 12.7 pg/ml, range 2.8 to 25.1 pg/ml), which reduced even more in 6 to 24 m DN RA (median 7.8 pg/ml, range 2.6 to 17.6 pg/ml). Serum IL-7 levels are reduced in RA independently of systemic levels of inflammation measured by CRP as previously demonstrated [10]. OA patients had normal IL-7 (median 13.1 pg/ml, range 9.7 to 19 pg/ml) and patients with other inflammatory conditions (including active colitis, psoriatic arthritis, ankylosing spondylitis, gout, reactive arthritis) showed reduced levels only in 14 out of 96 individuals (median 12.7 pg/ml, range 7 to 29.6 pg/ml). Further data on synovial IL-7 expression are presented in Figure S1 in Additional file 1.

Serum IL-7 was measured in 90 RA patients in CR (age range 23 to 79 years) with median remission duration of 18 months (range 6 to 72 months) and median disease duration of 115 months (range 36 to 300 months). Serum IL-7 levels in CR (Figure 1A) ranged from 2.5 to 22.9 pg/ml (median 10.7 pg/ml); 48% of patients had normal IL-7 levels (above 10 pg/ml as previously determined) [15,17].

### Lack of IL-7 recovery in CR is associated with relapse

Importantly, levels of IL-7 in CR were lower in patients who experienced a flare within an 18-month follow-up period (Figure 1B, left panel, *P* = 0.072). Amongst these patients, inability to recover healthy levels of IL-7 was observed in 13 out of 15 (86%) patients experiencing relapse within the follow-up period but only 11 out of 48 (23%) of those who did not (*P* = 0.0002). Demographic and clinical data were therefore investigated as to whether they could predict recovery of healthy levels of circulating IL-7 in RA patients in CR. Patient age or sex had no predictive value for recovering healthy IL-7 levels. There was no relationship between IL-7 levels and the duration of RA before achieving remission or the duration of remission itself. No relationship was observed between IL-7 and DAS28 score. Other clinical features of RA (RF positivity in 42% of patients, shared epitope in 70%, presence of erosions in 80% and extra-articular complications in 29%) were not associated with IL-7 levels in CR. Most patients achieved remission on therapy, but no association was found with current or past DMARDs (number or type) or the use of non-steroidal anti-inflammatory drugs. Extensive imaging was performed on this cohort and residual synovitis was detected in most patients either by ultrasound (78% of patients), power Doppler (45%) or magnetic resonance imaging (MRI) (96%) [12,41], but this revealed no association with IL-7 recovery.

Age at onset of RA influenced IL-7 recovery (Figure 1B middle panel; rho = 0.539, *P* = 0.001), suggesting that the earlier the disease occurred, the lower the likelihood of having normal levels of IL-7 during CR. Additional information was obtained related to family history, child bearing, oral contraception and smoking habits. Patients reported other cases of inflammatory arthritis (not medically confirmed as RA, but excluding OA) in their family only on the maternal side (mother, siblings, maternal aunt). Levels of IL-7 in this group of patients were significantly lower (Figure 1B right panel, *P* = 0.003). Non-smoking was rarely observed in this cohort (16% of patients), but was associated with an absence of recovery of healthy IL-7 levels (*P* = 0.042). Smoking at the time of disease onset was also weakly associated with lower levels of IL-7 (*P* = 0.063), compared to patients who had stopped smoking before the onset of RA. No differences in IL-7 were found between women who had or had not borne children, taken oral contraception or were post-menopausal at onset of disease. Binary regression was used to model IL-7 levels >10 pg/ml. This confirmed high significance for self-reported maternal family history of arthritis (OR = 7.66, *P* = 0.006) and possibly smoking (OR = 3.33, *P* = 0.068) with no further demographic or clinical dependency.

### T-cell differentiation and proliferative history

Differentiation of T cells between naïve, memory and Treg cells was investigated in relation to IL-7 in CR (n = 40). Naïve cell (CD4^+^CD45RB^high^CD45RA^+^CD62L^+^) frequency was quite variable but no relationship with age was observed as the recovery of thymic activity in CR is independent of age although related to levels of IL-7 [10]. In agreement with these data, naïve cell frequencies were weakly related to levels of IL-7 (n = 40, rho = 0.400, *P* = 0.010). In CR patients with normal of IL-7 levels (>10 pg/ml), the TREC content of naïve CD4^+^T cells (n = 15, median 6.5%, range 0.9 to 15.6%) was similar to an age-matched healthy control group (n = 15, median 7.1%, range 2.5 to 17.4%), confirming the absence of proliferation of naïve cells in remission. In CR patients with below normal levels of IL-7 (<10 pg/ml), the TREC content of naïve cells remained low (n = 13, median 2.57%, range 0.41 to 4.57%) suggesting absence of novel naïve-TREC+ cells being released.

IRCs were lower in remission (median 7.8% range 0.9 to 15.75%) compared to data in active RA (n = 40, median 10.5%, range 0.7 to 43%, not significant). In the group defined by healthy levels of IL-7 and associated with long-term remission, IRCs tended to be lower (median 3.6%, range 0.9 to 15.4) compared to the group with low IL-7 and increased risk of flare (median 9.6%, range 3 to 15.75, not significant). The frequency of IRCs was globally not related to serum IL-7 levels.

**Figure 2:**
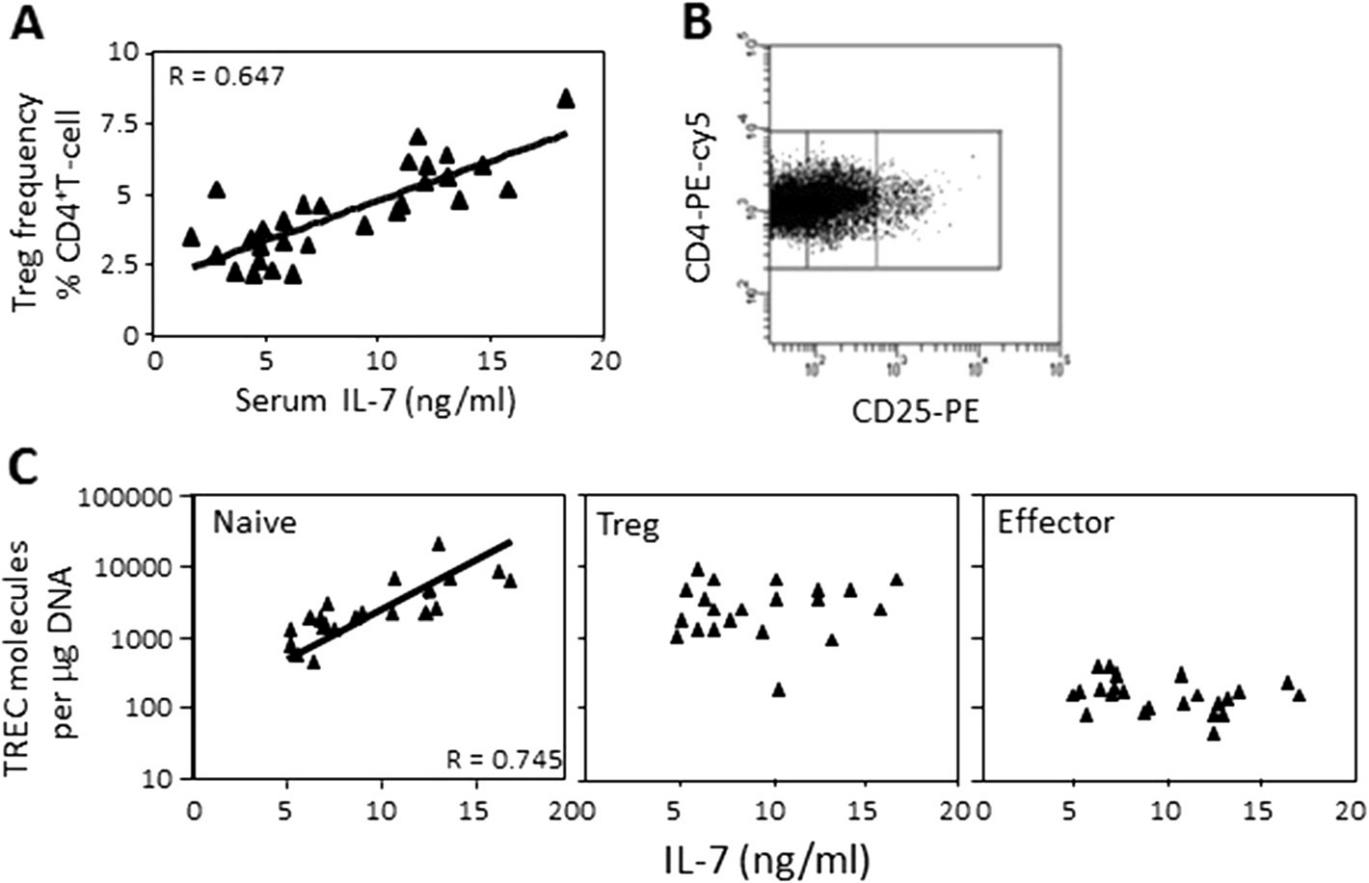
**IL-7 and Treg proliferative history. (A)** CD4^+^CD25^high^ T regulatory cells (Tregs) were quantified using flow cytometry in peripheral blood mononuclear cells (PBMCs) from 30 patients in clinical remission (CR) as a proportion of total CD4^+^ T cells. A direct correlation between the frequency of CD4^+^CD25^high^Tregs and serum interleukin (IL)-7 was observed (rho = 0.647, *P* <0.0001). **(B)** Flow cytometry strategy T cells (CD25^low^) and naïve T cells (CD25^−^ cells). The panel describes further the gating strategy used for cell sorting of naïve cells (bottom 30% of CD25^−^cells), effector cells (middle 30% of CD25^+^cells) and Tregs (top 75% of CD25^high^cells). **(C)** T-cell subsets were sorted from 21 CR patients using the gates described in Figure 2B. DNA was extracted and T-cell receptor excision circle (TREC) content measured by real-time PCR. TREC levels in both naïve and Treg subsets were high, suggesting a short proliferative history, but lower in the effector subset, indicating several rounds of proliferation. There was a positive correlation between *in vivo* IL-7 levels and TREC content of naïve T cells (rho = 0.745, *P* <0.002).

We previously showed that the frequency of Tregs (range 2.12 to 8.32% of total CD4^+^T cells) in CR was increased compared to active disease [11]. We re-analysed these data, in relation to serum levels of IL-7 and observed a direct correlation with the frequency of Tregs (Figure 2A, rho = 0.647, *P* <0.0001). The TREC content of the Treg subset was also used to measure the proliferative history of Tregs and compared to that of the effector T cells (CD25^low^) or naïve T cells (CD25^−^ cells). Flow cytometry strategy is described in Figure 2B. A direct relationship was confirmed between IL-7 levels and the TREC content of naïve T cells defined using this alternative phenotype (Figure 2C left panel, rho = 0.745, *P* = 0.002). As naïve T cells contain most TREC, this reproduced data showing direct relationship between circulating IL-7 and total CD4^+^T cell TREC content [10]. The TREC content of the Treg subset was high (Figure 2C middle panel, >1,000 molecules/μg DNA) suggesting absence of proliferation of Tregs. In contrast, the TREC content of effector T cells was low in all patients (Figure 2C right panel, <1,000 molecules/μg DNA), as these predominantly represent activated, effector and memory T cells, which had previously proliferated, reducing their TREC content.

### Stimulation by PHA

**Figure 3:**
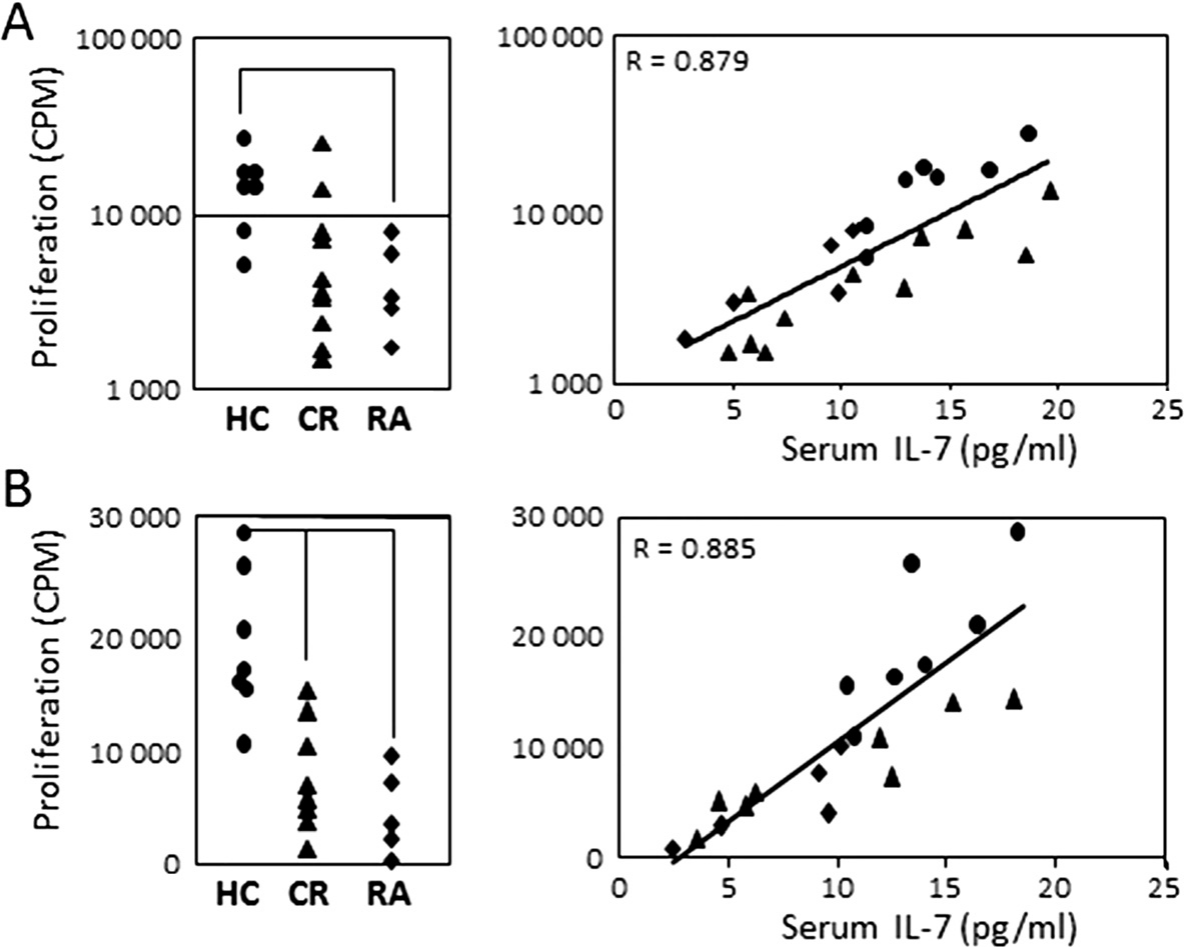
**Proliferation of T-cell subsets. (A)** Proliferation of total CD4^+^T cells in response to phyto-haemagglutinin (PHA) (2 μg/ml) in healthy control (HC) (n = 7, circles), active rheumatoid arthritis (RA) (RA, n = 5, diamonds) and in clinical remission (CR) (n = 11, triangles). Significantly better proliferation was observed in HC compared to active RA (*P* = 0.01) but not CR. A direct correlation was observed between proliferative responses of CD4^+^T cells and *in vivo* (serum) interleukin (IL)-7 (rho = 0.879, *P* <0.0001), combining all three groups. **(B)** Proliferation of effector T cells (CD4^+^CD25^−^) stimulated with PHA (2 μg/ml) in controls (n = 6, circles), active RA (RA, n = 5, diamonds) and CR (n = 8, triangles). Significantly better proliferation was observed in HC compared to RA (*P* = 0.003) and with CR (*P* = 0.001). A direct correlation was observed between serum IL-7 and effector cell proliferation (rho = 0.885, *P* <0.0001) combining the three groups.

The response of CD4^+^T cells (negatively separated on magnetic beads) to PHA stimulation was measured in relation to serum IL-7 levels. We observed high levels of proliferation in HC (Figure 3A) compared to DN early RA patients (*P* = 0.01). Proliferation in the CR patients was variable. The *in vivo* levels of exposure to IL-7 were positively correlated with the response of CD4^+^T cells to PHA stimulation (Figure 3A, rho = 0.879, *P* <0.0001). Spontaneous proliferation was negligible and there was no difference between groups or in relation to IL-7 levels (data not shown).

We isolated CD4^+^Tregs (using the same strategy as in Figure 2B) and effector T cells by cell sorting. Proliferation of CD4^+^CD25^−^T cells in response to PHA was significantly higher in controls than in active RA (Figure 3B, *P* <0.001) or in CR (*P* = 0.003). The proliferative capabilities of effector CD4^+^CD25^−^T cells in response to PHA were also directly related to serum levels of IL-7 (Figure 3B, rho = 0.885, *P* <0.001). In contrast, CD4^+^Tregs responded poorly to PHA alone regardless of clinical group (data not shown, less than 3,000 CPM).

### Co-stimulation with IL-7

**Figure 4:**
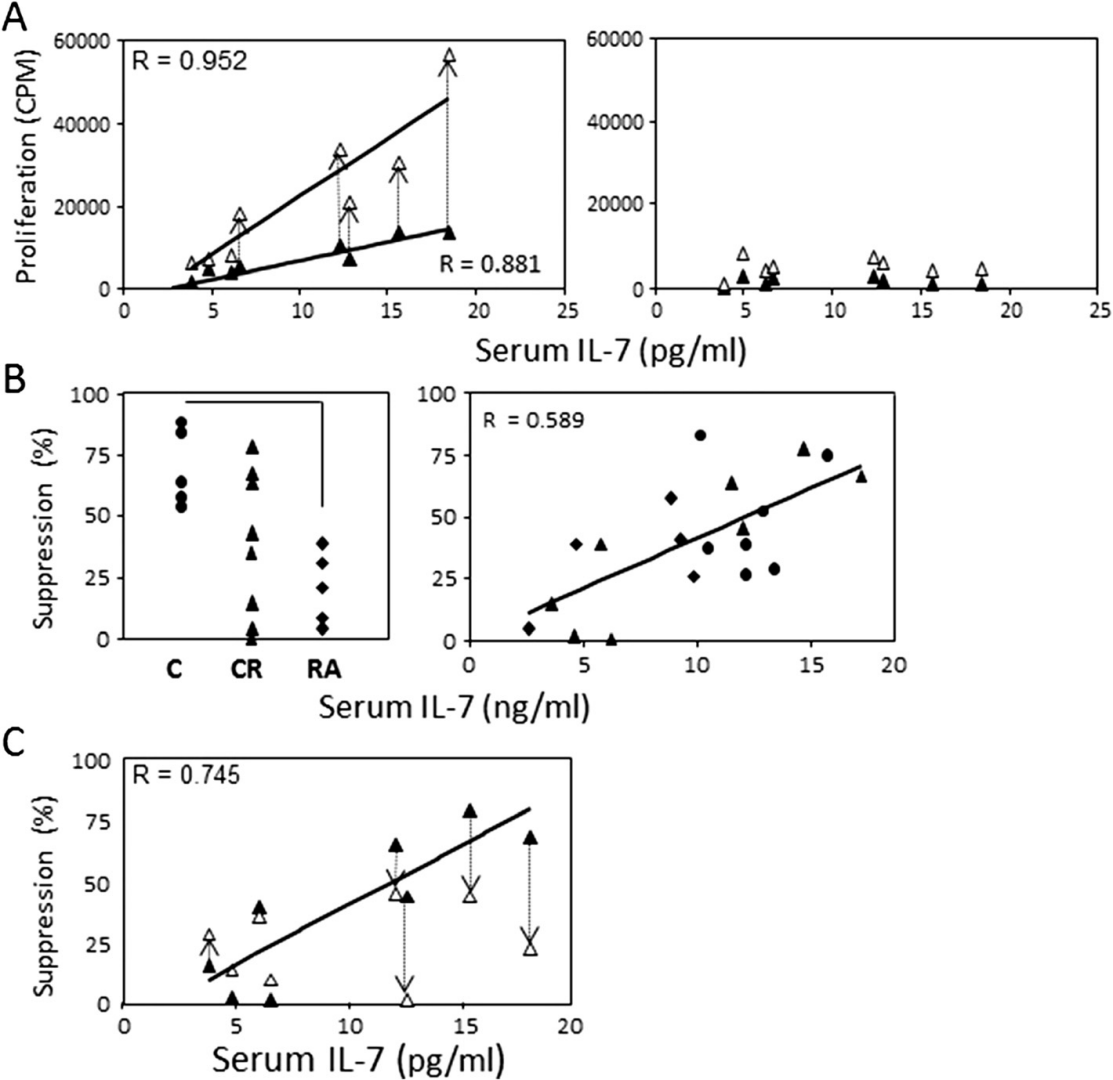
**Proliferation and suppression with IL-7 co-stimulation. (A)** Proliferation of CD4^+^CD25^−^ effector T cells (left panel) and CD4^+^CD25^high^T regulatory cells (Tregs) (right panel) in response to phyto-haemagglutinin (PHA) alone (1 μg/ml, closed symbols) and in the presence of interleukin (IL)-7 (10 ng/ml, open symbols) was measured in eight clinical remission (CR) patients. Dotted arrows indicate changes in proliferative activity. The direct correlation between effector T-cell proliferation and serum IL-7 (rho = 0.952, *P* <0.0001) was maintained in the presence of co-stimulation with IL-7 (rho = 0.881, *P* = 0.004). There was a marginal increase in the proliferation of CD4^+^CD25^high^Tregs in the presence of IL-7 but proliferation responses remained very low. **(B)** CD4^+^CD25^high^Treg and CD4^+^CD25^−^T cells were co-cultured in the presence of PHA (2 μg/ml). Percentage suppression was measured in controls (n = 5, circles), active rheumatoid arthritis (RA) (n = 5, diamonds) and CR (n = 8, triangles). Suppression was significantly reduced in RA (*P* <0.0001). A direct trend was observed between suppression capabilities and serum IL-7 levels (rho = 0.589, *P* = 0.006). **(C)** The suppression assay was repeated in the presence of IL-7 co-stimulation (10 μg/ml) for the eight patients in CR. Dotted arrows indicate changes in suppression activity. Suppression was marginally affected in four patients with low IL-7 serum levels (<10 pg/ml) but reduced to a greater degree in patients with normal serum IL-7 levels.

Co-stimulation with IL-7 enhances T-cell responses to stimuli such as PHA, anti-CD3/CD28 or purified protein derivative [15]. The proliferation assays using effector/Treg cells were repeated in the presence of IL-7 (10 μg/ml) as co-stimulator with PHA activation in CR patients. IL-7 alone did not elicit any proliferative response (data not shown). Effector CD4^+^T cells showed enhanced proliferation in response to PHA + IL-7 (Figure 4A left panel, n = 8, rho = 0.952, *P* <0.0001; open symbols) maintaining the relationship with serum IL-7 levels observed in the absence of co-stimulation (symbols filled). However, this effect appears to be much more pronounced in patients with normal *in vivo* levels of IL-7 whereas those lacking IL-7 showed minimum improvement of proliferation. In Tregs, proliferation was marginally increased by the co-stimulation with PHA + IL-7 and the lack of relationship with IL-7 was maintained (Figure 4A, right panel).

### Suppression

We next investigated whether suppression of effector T-cell proliferation by CD4^+^Treg (in response to PHA) was related to *in vivo* IL-7. Suppressive capacity was significantly higher in cells from HC (Figure 4B, left panel) than those from active RA patients (*P* <0.05). Suppression in CR was highly variable and directly related to serum IL-7 levels (Figure 4B, rho = 0.589, *P* = 0.006).

Co-stimulation with IL-7 during suppression of effector T cells by Tregs *in vitro* was shown to abolish Treg-suppressive activity [42,43]. The results of the suppression assay performed in the presence of PHA + IL-7 were dichotomous: in CR patients with low *in vivo* IL-7, minimal variation in suppressive activity was observed (Figure 4C). In contrast, in CR patients with normal serum IL-7, reduction of suppression was observed in the presence of exogenous IL-7 (10 to 30% less suppression, indicated by arrows).

### Survival

We first examined IL-7R expression using flow cytometry on Tregs and T cells (CD127-MFI; Figure S2A in Additional file 1). We observed minimal difference between groups (Figure S2B in Additional file 1) suggesting that changes in T-cell response to IL-7 are unlikely to be due to difference in levels of receptor expression although expression does vary (by a factor of approximately 2) at the protein level within each clinical group.

IL-7 has the potential to alter cell survival through the regulation of BCL2, a key anti-apoptotic protein [44]. To determine whether improved T-cell responses were related to changes in the apoptotic signalling balance between BCL2 and BAX, real-time PCR was used to measure *BCL2* and *BAX* expression in cells from HC, active RA and CR patients. Expression levels were not significantly different between any of the three groups (Figure S3 in Additional file 1, *P* >0.750 for each comparison).

Furthermore, there was no relationship between *BCL2* or *BAX* expression and serum IL-7 (rho <0.01), suggesting that differences in IL-7 were not sufficient to alter the balance of pro- and anti-apoptotic factors and therefore do not improve response through increased viability of cells.

### Relationship of IL-7 with other cytokines

**Figure 5:**
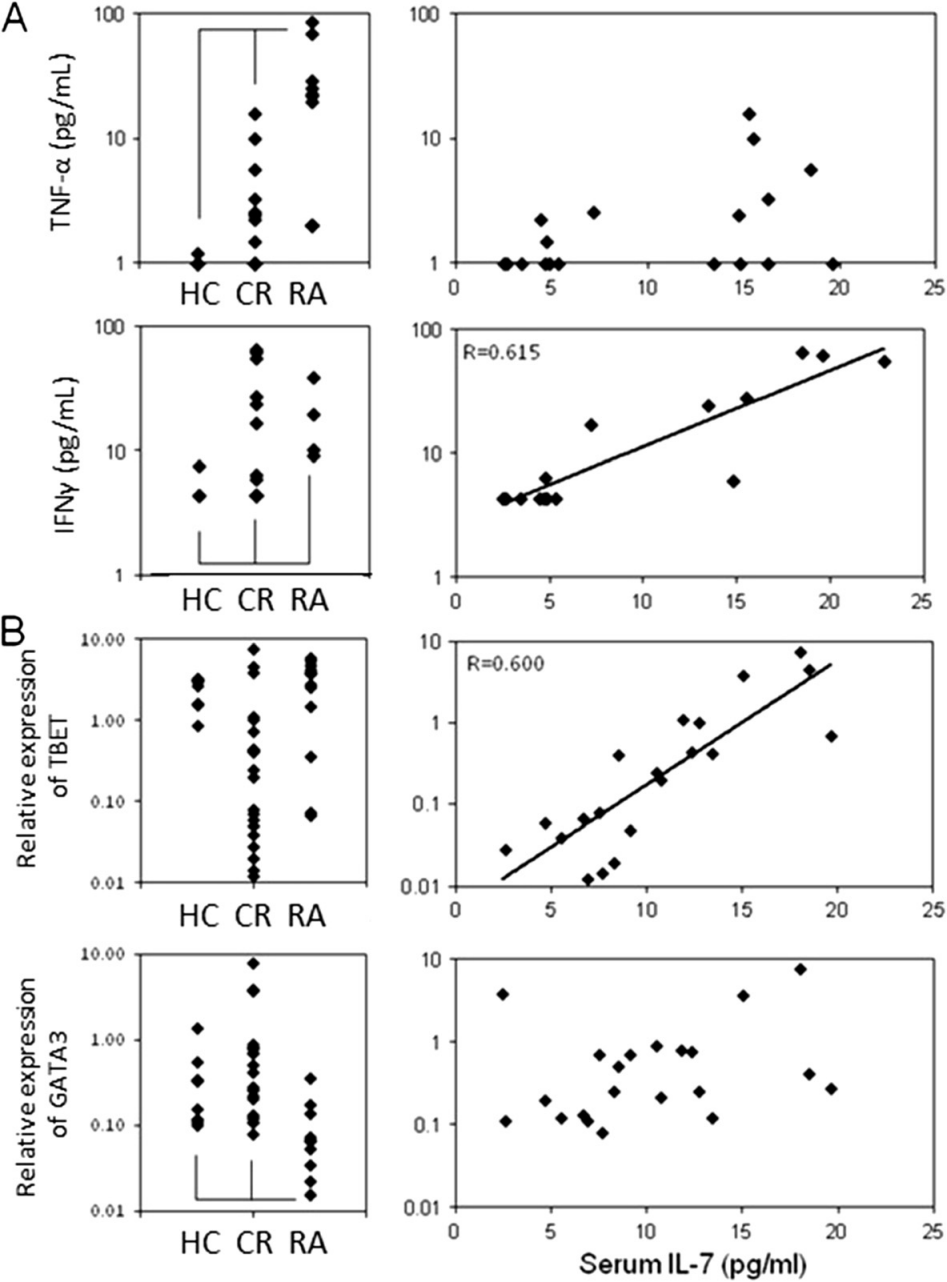
**Cytokine network and polarization. (A)** Circulating levels of tumour necrosis factor (TNF)-α and interferon (IFN)-γ were measured by enzyme-linked immunosorbent assay (ELISA) in serum from healthy control (HC) (n = 8), active rheumatoid arthritis (RA) (n = 10) and clinical remission (CR) (n = 20) patients. Levels were mostly below detection in HC (with the exception of two individuals). Increased levels of circulating cytokines were observed in active RA (*P* <0.05). In CR, a direct relationship was observed between interleukin (IL)-7 and IFN-γ (rho = 0.615, *P* = 0.005) but not with TNF-α. **(B)** Expression of *TBET* and *GATA3* was measured by real-time PCR in cells from HC (n = 8), active RA (n = 10) in CR (n = 20). No significant changes were seen in *TBET* expression, however, *GATA3* expression was lower in RA compared to HC (*P* = 0.0172) and CR (*P* = 0.0002). No difference was observed between HC and CR. In CR, a direct relationship was observed between serum IL-7 and *TBET* expression (rho = 0.600, *P* = 0.011) but not with *GATA3*.

Cytokines often work in a network, their expression regulating each other. Possible interplay between IL-7 and other cytokines (IL-2, IL-6, IFN-γ, TNF-α, TGF-β1) was investigated in sera from HC, active RA patients and CR. Low levels of circulating cytokines were observed in HC (below detection for most individuals), which significantly differed from levels measured in active RA (Figure 5A, *P* <0.05 for all cytokines, data not shown for IL-2, IL-6 and TGF-β1). In CR, a direct relationship was observed between IL-7 and IFN-γ (Figure 5A rho = 0.615, *P* = 0.005). In contrast, all other cytokine levels were independent of IL-7 (Figure 5A for TNF-α and data not shown for IL-6 and TGF-β1).

### Th1/Th2 polarization

The exact pathogenesis of RA remains unclear, although it is often referred to as a Th1-driven disease. In relation to the role of IL-7 in Th1 polarization, the commitment of cells towards Th1 was further investigated by measuring their expression of *TBET (TBX21)* and *GATA3*, two transcription factors essential for Th1 and Th2 polarization, respectively [45]. There was no significant difference in expression of *TBET* (Figure 5B) but in CR *GATA3* expression was comparable to HC, both being significantly higher than RA (*P* <0.020). In CR, a direct relationship was observed between serum IL-7 and *TBET* expression (Figure 5B rho = 0.600, *P* = 0.011) but not with *GATA3*.

## Discussion

We examined a cohort of RA patients in CR (with low clinical and biochemical disease activity) in whom the potentially confounding influence of inflammation was minimized and compared them with HC and patients with active RA. A total of 48% of RA patients in CR had normal *in vivo* circulating levels of IL-7. Lack of IL-7 recovery in CR was associated with increased risk of relapsing and related to familial history of arthritis and possibly smoking. Serum IL-7 in CR varied from normal healthy levels to the low levels observed in active RA [10,15,17]. We confirmed the absence of stimulatory effect of IL-7 alone on T-cell proliferation [10] as well as showing the same lack of effect on Tregs. Normal circulating levels of IL-7 were associated with improved T-effector but not Treg responses to PHA stimulation across clinical groups. Most importantly, the suppressive capabilities of Tregs were directly related to *in vivo* exposure to IL-7. In contrast, *in vitro* co-stimulation with PHA + IL-7 particularly enhanced the proliferation of effector T cells with minimal effect on Tregs. Suppression in the presence of PHA + IL-7 was reduced in CR patients with normal *in vivo* levels of IL-7. The extent of Th1 polarization was also directly related to *in vivo* exposure to IL-7. None of these effects appear to be mediated by a change in expression of survival factor or the degree of IL-7R expression on T-cell subsets. We could not relate any of these findings to previous or current DMARD usage.

There are several theories regarding the relationship between circulating IL-7 levels and T cells. According to one hypothesis, the amount of circulating IL-7 depends on the rate of its consumption by T cells [46]. This has been confirmed in conditions including cancer and HIV infection, but not in RA where lymphodepletion did not lead to IL-7 accumulation [10]. An alternative hypothesis suggests that ‘altruism’ could regulate T-cell proliferation; having satisfied their IL-7 requirement, a number of T cells would abstain from consuming IL-7 by downregulating IL-7R expression [46]. Our data showing no differences in levels of IL-7R expression on T cells do not support this hypothesis in RA (at least compared to HC or CR). Further hypotheses explaining the regulation of circulating IL-7 levels in RA are therefore required. It is however, established that circulating soluble-IL-7R (sIL-7R) may have a role [47,48]. Recently, inflammation was shown to increase sIL-7R expression in systemic lupus erythematosus and diabetes mellitus type 1 [49]. In multiple sclerosis, aberrant splicing of exon 6 was shown to increase the production of *sIL-7R* [50–52] and that sIL-7R is able to potentiate IL-7 bioactivity and to promote autoimmunity [53]. We investigated levels of sIL-7R in 20 CR patients (Figure S4 in Additional file 1) and showed no direct relationship with levels of IL-7, suggesting that future work towards understanding the regulation of circulating IL-7 in RA is still needed.

The lack of IL-7 recovery in CR could indicate low levels of disease activity (eventually leading to sIL-7R expression keeping circulating levels low), however we could not establish any relationship between IL-7 levels and residual disease activity even when advanced ultrasound and MRI techniques were used [10,41,54]. We observed associations with smoking and early disease onset. Serum IL-7 was identified as a diagnostic marker in head and neck cancer [55]. Exposure to cigarette smoke elicited an increase in serum IL-7 in mice lacking the multidrug resistance genes [56]. Thus, associations between IL-7 and smoking have been reported but further work is needed to understand the mechanisms behind these and our own observations. The strongest association observed was with a self-reported maternal family history of arthritis suggesting a genetic link, although recent data provided contradictory evidence with respect to associations between *IL-7R* polymorphisms and RA [57–60].

Our data showing a direct relationship between Treg frequency and circulating *in vivo* IL-7 levels are in agreement with previous reports of a similar role in regulating Treg homeostasis in mice [61]. Contrary to mice, our data appear to limit this effect to thymic release of new Tregs in humans rather than through both homeostatic proliferation and thymic activity. IL-7 signalling through STAT-5 was also shown to regulate the expression of Foxp3 in human cells [62,63], which may explain the enhanced suppressive capacity of Tregs in relation to IL-7. *In vitro* priming of mouse Tregs with IL-7 increased their suppressive functions [42], which also support our findings in humans. Co-stimulation with IL-7 also appears to have effects depending on priming by IL-7, which may explain the discrepancies in T-cell behaviour between blood (anergy) and synovium/synovial fluid in RA (hyper-reactivity) also rendering cells insensitive to suppression by Tregs.

IL-7 was shown to favour Th1 polarization via a direct effect on DC [64,65] and T-cell expression of the IL-12R [66]. The mechanism by which IL-7 polarizes DC into DC1 is yet to be elucidated [67] but Th1 polarization is affected in RA [49,68,69] and defects in *TBET* mRNA expression were reported [45,70]. Our data therefore suggest that the deficit in IL-7 in RA may contribute to the qualitative defect in Th1 polarization. An implication of IL-7 in Th17 differentiation has recently been proposed in primary Sjögren’s syndrome and in skin cancer [20,71] although it remained to be explored in RA. An unstable commitment towards Th1 polarization may therefore favour the development of Th17 cells [72] and such bias may result in chronicity.

Further to its direct effect on thymic release of new T cells and polarization, we found strong correlations between *in vivo* exposure to IL-7 in an individual and their T-cell responsiveness to PHA (and also to TCR or recall antigen, data not shown) [15] and suppressive capabilities. A missing signal provided by IL-7 could also explain this effect. IL-7 is a major anti-apoptotic/survival factor for T cells through its upregulation of BCL-2 [44,73] and T cells deprived of IL-7 die within a few days [46,74]. Our data suggest that the lack of direct exposure to IL-7 *in vivo* was not associated with any detectable effect on the balance between pro- and anti-apoptotic factors. Furthermore, patients had normal T-cell numbers across the range of IL-7 levels (data not shown). It is therefore unlikely that a deficit in survival signals could explain our observations on responsiveness and suppression. The alternative explanation that IL-7 provided a ‘priming’ signal that influences T-cell behaviour remains the most attractive one [21]. The mechanism of priming, by which cells maintain long-term memory of a signal that modifies their behaviour at a later point in time (that is polarization, transition from naïve to memory), is not clear; however IL-7 was reported to induce long-term chromatin remodelling via STAT-5 [75]. It could be hypothesized that IL-7 exposure causes additional long-term effects through a similar mechanism.

## Conclusions

Altogether, our data suggest that variation in IL-7 levels may explain circulating T-cell dysfunctions in RA [4,16]. The difference in systemic and joint T-cell behaviours may also be related to differences in levels of IL-7. Although ethical limitation prevented access to synovial T cells and collection of SF in CR, we have shown that IL-7 expression was reduced in synovial tissue biopsies in relation to local inflammation although these patients were not in CR (Additional file 1). We have previously discussed the discrepancy between low systemic IL-7 and high synovial expression in relation to progression of bone erosion suggesting that reduced systemic IL-7 is an indicator of active synovitis [17]. Low systemic IL-7 in CR may therefore suggest persistent high joint IL-7, despite no indication that circulating IL-7 levels in CR were linked to residual disease activity detected by qualitative imaging technologies. The association between relapse and lack of IL-7 recovery may suggest that the mechanism controlling IL-7 recovery remains in ‘active disease mode’ in such patients and that CR may not be equivalent to immunological remission as much as it does not equate to imaging remission [41]. In conclusion, our data reinforce that in humans, the concept of blocking IL-7 in autoimmunity may have important therapeutic effects, as recently demonstrated in the collagen-induced arthritis model [76] as well as in several other autoimmune disease animal models [77–82] and in melanoma [71]. Two clinical trials in multiple sclerosis (NCT02045732) and diabetes mellitus type 1 (NCT02038764) using an antibody that binds to and inhibits the human IL-7 receptor are actively recruiting participants.

## Additional file

Additional file 1:
**Method: Immunohistochemistry and digital imaging scoring.** **Table S1.** Presents the demographic data of the cohorts studied. **Figure S1A.** Describes synovial fluid levels of IL-7 measured in RA (n = 32), OA (n = 25) and reactive arthritis (n = 4). **Figure S1B.** Presents immunohistochemistry staining for IL-7 expression in synovial biopsies from RA (n = 25) and OA (n = 5) patients and results of digital image analysis used to score IL-7 expression. **Figure S1C.** Shows correlation of IL-7 expression with arthroscopic VAS. **Figure S2A.** Describes IL-7R (CD127) expression on the cell surface of CD4 + T-cell subsets. **Figure S2B.** Shows surface expression of IL-7R on T cells and Tregs in HC (n = 78), early RA (ERA, n = 50), 24 long-lasting RA (RA, n = 24) and CR (n = 26). **Figure S3.** Presents expression of BCL2 and BAX measured by real-time PCR in HC (n = 8), active RA (n = 10) and in CR (n = 18). **Figure S4.** Lack of correlation between sIL-7R to IL-7.

## Abbreviations

ACPA: anti-citrullinated protein antibody
CR: clinical remission
CRP: C-reactive protein
DAS: disease activity score
DC: dendritic cell
DMARD: disease-modifying anti-rheumatic drugs
DN: DMARD-naïve
ELISA: enzyme-linked immunosorbent assay
HC: healthy control
IFN: interferon
IL: interleukin
IRCs: inflammation-related cells
MFI: mean fluorescence intensity
MHC: major histocompatibility complex
MRI: magnetic resonance imaging
MTX: methotrexate
NK: natural killer
OA: osteoarthritis
OR: odds ratio
PBMCs: peripheral blood mononuclear cells
PCR: polymerase chain reaction
PHA: phyto-haemagglutinin
RA: rheumatoid arthritis
RF: rheumatoid factor
SF: synovial fluid
SFMCs: synovial fluid mononuclear cells
sIL-7R: soluble interleukin-7 receptor
TGF: transforming growth factor
Th: T helper cell
TNF: tumour necrosis factor
TRECs: T-cell receptor excision circles
Tregs: regulatory T cells
