# Supplementary material for: Modulation of peripheral T-cell function by interleukin-7 in rheumatoid arthritis
Source: Arthritis Res Ther. 2014 Dec 23;16(6):511. doi: 10.1186/s13075-014-0511-3 (PMC4298067; doi:10.1186/s13075-014-0511-3)
Supplement: Additional file 1: — Method: Immunohistochemistry and digital imaging scoring. Table S1. Presents the demographic data of the cohorts studied. Figure S1A. Describes synovial fluid levels of IL-7 measured in RA (n = 32), OA (n = 25) and reactive arthritis (n = 4). Figure S1B. Presents immunohistochemistry staining for IL-7 expression in synovial biopsies from RA (n = 25) and OA (n = 5) patients and results of digital image analysis used to score IL-7 expression. Figure S1C. Shows correlation of IL-7 expression with arthroscopic VAS. Figure S2A. Describes IL-7R (CD127) expression on the cell surface of CD4 + T-cell subsets. Figure S2B. Shows surface expression of IL-7R on T cells and Tregs in HC (n = 78), early RA (ERA, n = 50), 24 long-lasting RA (RA, n = 24) and CR (n = 26). Figure S3. Presents expression of BCL2 and BAX measured by real-time PCR in HC (n = 8), active RA (n = 10) and in CR (n = 18). Figure S4. Lack of correlation between sIL-7R to IL-7. [file 13075_2014_511_MOESM1_ESM.docx]

# **Additional File**

# **Modulation of peripheral T-cell function by interleukin-7 in Rheumatoid Arthritis.**

Sarah M Churchman^1^, Jehan J El-Jawhari^1^, Agata N Burska^1^, Rekha Parmar^1^, Vincent Goëb^2^, Philip G Conaghan^3^, Paul Emery^3^ and Frederique Ponchel^1*^

^1^Leeds Institute of Rheumatic and Musculoskeletal Medicine, Leeds Musculoskeletal Biomedical Research Unit, St James’s University Hospital, Beckett St, Leeds, LS9 7TF, UK

^2^Rheumatology Department, University Hospital of Amiens, University of Picardie Jules Verne, 80000 Amiens, France

^3^Leeds Institute of Rheumatic and Musculoskeletal Medicine, Leeds Musculoskeletal Biomedical Research Unit, Chapel Allerton Hospital, Chapeltown Road, Leeds, LS7 4SA, UK

^*^ Corresponding author: Frederique Ponchel

Clinical Sciences Building,

St. James's University Hospital,

LEEDS, LS9 7TF, UK

Tel. + 44 (0)113 2065642

E-mail mmefp@leeds.ac.uk

We report no conflict of interest.

This additional file contains:

Method

*Immunohistochemistry and digital imaging scoring*

Additional Figures

*Additional Table 1 –* Presents the demographic data of the cohorts studied.

*Additional Figure 1A* - describes synovial fluid levels of IL-7 measured in RA (n=32), OA (n=25) and reactive arthritis (n=4).

*Additional Figure 1B* - presents immunohistochemistry staining for IL-7 expression in synovial biopsies from RA (n=25) and OA (n=5) patients and results of digital image analysis used to score IL-7 expression.

*Additional Figure 1C* - shows correlation of IL-7 expression with arthroscopic VAS.

*Additional Figure 2A* - describes IL-7R (CD127) expression on the cell surface ofCD4+T-cell subsets.

*Additional Figure 2B* - shows surface expression of IL-7R on T-cells and Treg in HC (n=78), early RA (ERA, n=50), 24 long lasting RA (RA, n=24) and CR (n=26).

*Additional Figure* *3* - presents expression of *BCL2* and *BAX* measured by real-time PCR in HC (n=8), active RA (n=10) and in CR (n=18).

*Additional Figure 4* - Lack of correlation between sIL-7R to IL-7

*Immunohistochemistry and digital imaging scoring*

Slides from paraffin embedded sections were de-waxed in Access Super solution (Menarini diagnostics) before being washed and stained. Slides were incubated in peroxidase block buffer followed by Casein block solution (Menarini diagnostics) for 10 minutes each. Incubation in IL-7 mouse monoclonal antibody at dilution 1:200 (R&D labs, MAB207) was performed for 1 hour followed by 3 washes. The Universal probe reagent was applied for 30 minutes and then X-Cell Polymer HRP reagent (Menarini diagnostics) for 30 minutes prior to adding of 3’-Diaminobenzidine (DAB) solution for 5 minutes. Slides were counterstained in haematoxylin for 2 minutes, then dehydrated through ethanol and mounted in Di-N-Butyle Phthalate Xylene.

Slides were examined under bright-field mode using a multispectral Nuance camera (Caliper, PerkinElmer) operated using the CRi Nuance V.3.0.1.2 software. A series of images were captured using 20 x magnifications with multispectral light scanning (420-720 nm). The Nuance software was capable of differentiating between the counter-stain spectral image (haematoxylin) and the immuno-stain image (DAB). The analysis was performed using the inForm V.1.4 software (CRi). The application was first trained to recognise three classes of tissues: cellular regions, vessels and fatty areas, by exemplifying representative areas and repeated until an accuracy of > 95% was obtained. Folds in the tissue or air bubbles were manually edited. The second step delineated each cell by means of segmentation between cytoplasm and nucleus in cellular areas only. Quantification of IL-7 was performed using a threshold in the optical density of staining using 256 shades of DAB: negatively stained cells including some background or false positive staining and cells and area of IL-7 diffusion considered positive (above shade 50). The final scores represent the percentage of positive cells and region of IL-7 diffusion excluding area covered by blood vessels and a-cellular regions of the whole biopsy surface.

*Additional Table 1* Demographic data of the cohorts studied (data are presented as median and range)

|  | HC | <6m DN RA | 6-24m DN RA | RA | CR | OA | others |
| --- | --- | --- | --- | --- | --- | --- | --- |
| n | 80 | 127 | 37 | 55 | 90 | 19 | 96 |
| Sex (% female) | 48 | 66 | 64 | 69 | 77 | 75 | 65 |
| Median age  (Years) | 44  (24-68) | 48  (21-80) | 52  (24-78) | 53  (23-81) | 51  (23-79) | 52  (27-77) | 51  (19-80) |
| Disease duration  (months) | NA | <6 | 6-24 | >60 | 115  (36-300) | NA | NA |
| Remission duration  (months) | NA | NA | NA | NA | 18  (6-72) | NA | NA |

DN = DMARD naïve, NA= not applicable

**
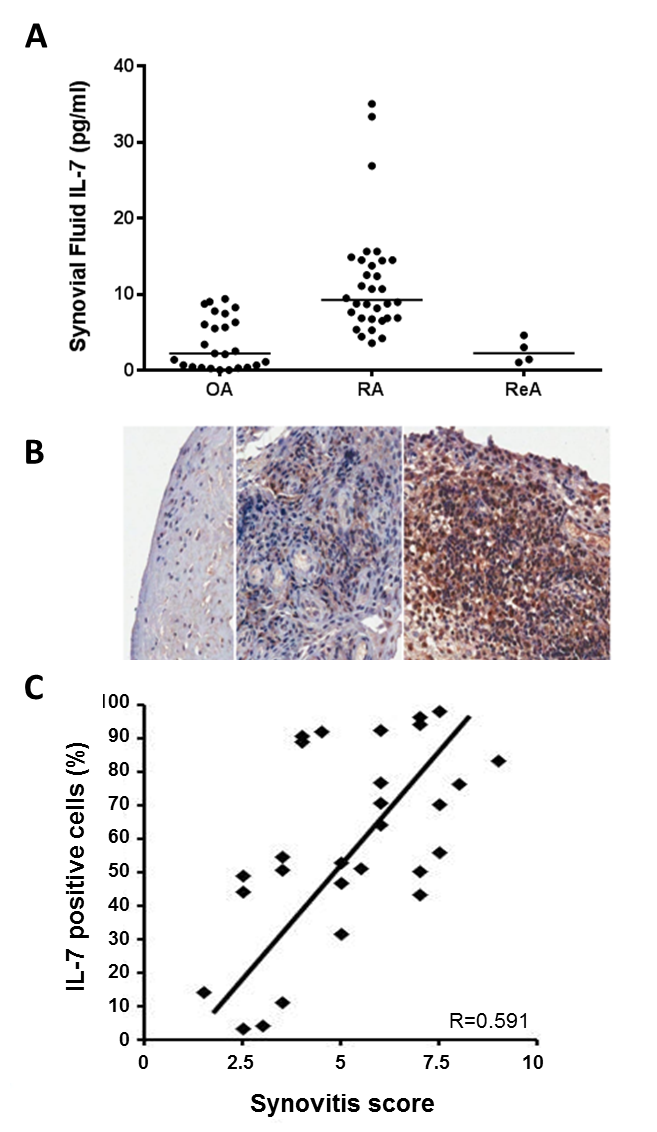
**

**Additional Figure 1. IL-7 measures in controls and RA patients**

A) Synovial fluid IL-7 levels in active RA (n=32) were significantly higher than in OA (n=25, p<0.05). IL-7 levels in reactive arthritis fluid were low (ReA, n=4).

B) Expression of IL-7 was detected using immunohistochemistry. Discrete positive cells expressing IL-7 were observed (middle panel, 100 x magnification) as well as regions of expression with both positive cells and extracellular diffusion (right panel 100 x magnification) (negative control antibody, left panel 200 x magnification).

C) IL-7 expression (digital imaging score) showed correlation with inflammation measured by arthroscopic visual inspection (VAS; rho=0.591, p=0.001).

**
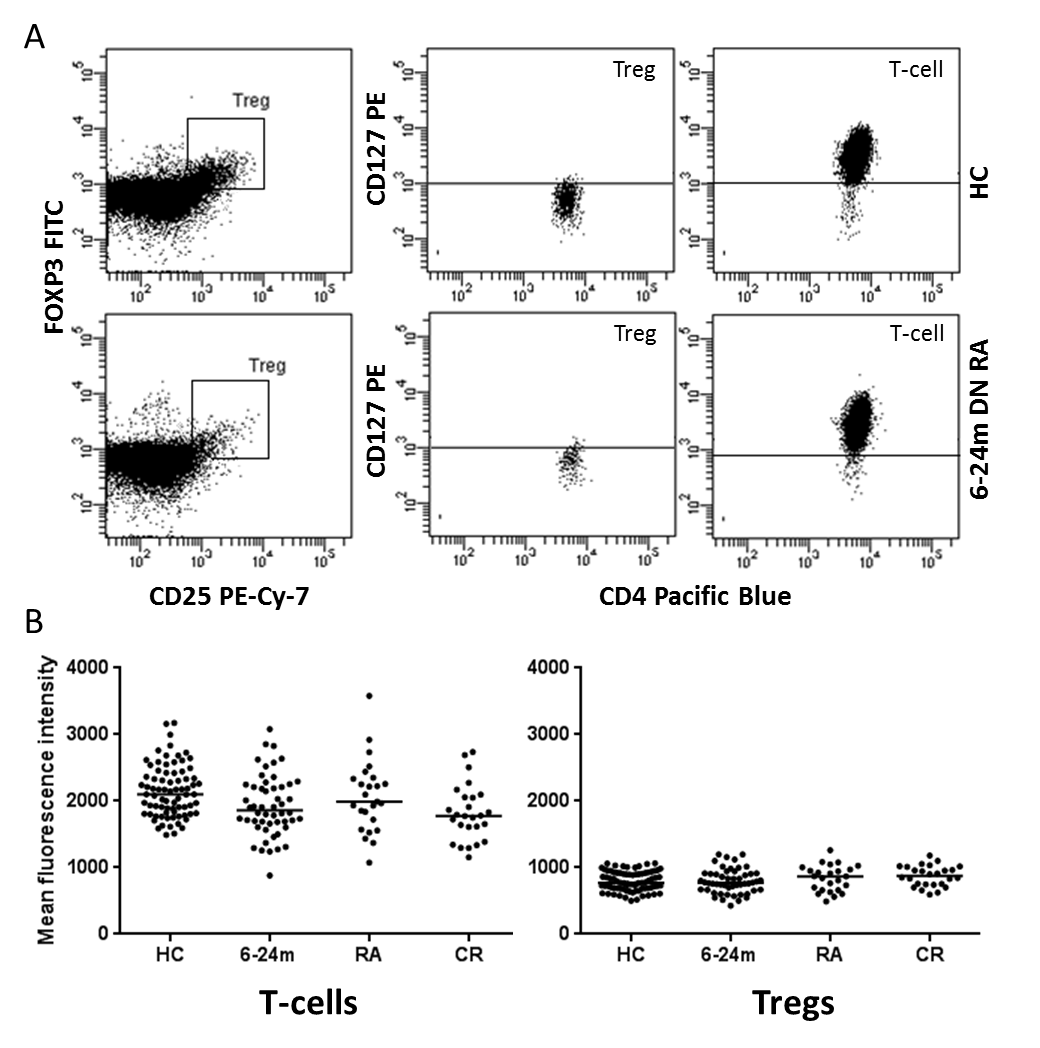
**

**Additional Figure 2. Expression of the IL-7 receptor**

A) Flowcytometry was used to measure IL-7R (CD127) expression on the cell surface ofCD4^+^T-cell subsets. Tregs were gated on the expression of CD25^high^ and Foxp3^+^ and the expression of IL-7R/CD127 was measured on both Tregs and the remaining T-cells.

B) Surface expression of IL-7R on T-cells and Treg in HC (n=78), 6-24m DMARD naïve RA (6-24m, n=50), 24 long lasting RA (RA, n=24) and CR (n=26). No significant differences were found in CD127 expression between any groups

**
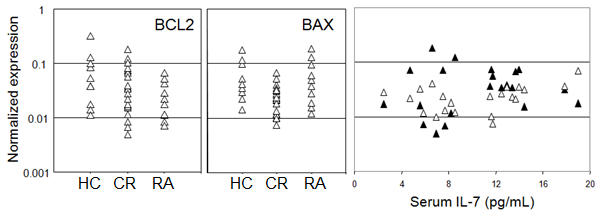
**

**Additional Figure 3 Expression of *BCL2* and *BAX***

Expression of *BCL2* (black) and *BAX* (white) measured by real-time PCR in HC (n=8), active RA (RA, n=10) and in CR (n=21). No difference was observed between any of the groups.

**Additional Figure 4 Lack of correlation between sIL-7R to IL-7**

Levels of IL-7 and sIL-7R were tested in patients in CR (n=20). No correlation was observed (rho=0.01). A commercially available ELISA (Human Soluble Interleukin-7 Receptor ELISA Kit, CUSABIO, , Upper Heyford, UK) was used to measure sIL-7 levels.
